# Ultrasound-guided thrombin injection for treatment of superficial traumatic pseudoaneurysms and associated expanding hematomas: experience in five patients

**DOI:** 10.1186/s13032-016-0034-9

**Published:** 2016-02-23

**Authors:** Sri Hari Sundararajan, Phillip Murillo, Adam Khan, Vyacheslav Gendel, Christopher Gribbin, Sudipta Roychowdhury, John Nosher

**Affiliations:** Department of Radiology, Rutgers-Robert Wood Johnson Medical School, New Brunswick, NJ USA; Rutgers-Robert Wood Johnson Medical School, New Brunswick, NJ USA; University Radiology Group, East Brunswick, NJ USA

**Keywords:** Thrombin, Pseudoaneurysm, Superficial, Hematoma, Ultrasound

## Abstract

**Background:**

Angiography allows for excellent characterization and treatment of traumatic pseudoaneurysms. However, ultrasound-guided thrombin injection for pseudoaneurysm thrombosis allows for radiation-free treatment of superficial pseudoaneurysms and superficial expanding hematomas.

**Methods:**

A retrospective review of 5 patient cases treated under this paradigm was performed following institutional review board approval. Outcomes following intervention were recorded and compared amongst the patient cohort.

**Results:**

Ultrasound-guided closure of traumatic pseudoaneurysms allowed for reduced procedural times and procedural invasiveness.

**Conclusions:**

As demonstrated by the following cases, ultrasound guided thrombin injection is a good method of primary treatment for superficial pseudoaneurysms, or as an alternative treatment in cases where transcatheter embolization fails.

## Findings

Angiography allows for excellent characterization and treatment of vascular pathology in the traumatic setting. While the medical necessity for fluoroscopy cannot be overemphasized, radiation awareness has become an important subject in the medical community. Campaigns such as Image Wisely and Image Gently have made great strides in establishing the importance of minimizing radiation exposure when medically allowable and without sacrificing therapeutic efficacy.

Cope and Zeit introduced the use of thrombin for inducing thrombosis of pseudoaneurysms in the medical literature as an effective therapeutic strategy in 1986 [1]. Thrombin, a derivative of prothrombin, assists in the cleavage of fibrinogen to fibrin, thus resulting in thrombus formation. Exogenous bovine-derived thrombin, which is used in clinical practice, leads to an identical mechanism causing the cleavage of fibrinogen [2]. Ultrasound interrogation is effective in confirming the “to and fro” Doppler pattern of pseudoaneurysms, visualizing needle tip entry into the pseudoaneurysm during therapeutic intervention, and surveying the immediate vicinity for additional sites of potential vascular injury [3].

The clinical utility of ultrasound-guided thrombin injection has been documented regarding inducing therapeutic thrombosis in superficial femoral artery pseudoaneurysms [4–7]. Additional clinical scenarios for implementation of this technique have also been documented, ranging from treatment of visceral pseudoaneurysms to superficially located arteries prone to pseudoaneurysm formation [8–10].

A retrospective review of 5 non-SFA pseudoaneurysm patients treated between July and November 2015 under this paradigm was performed following institutional IRB approval. Implementation of this technique in the emergent traumatic setting was expected to reduce procedural times and procedural invasiveness. The following exclusion criteria were implemented prior to consideration for this protocol: rapid expansion of the pseudoaneurysm upon arrival to the emergency department, clinically significant pseudoaneurysm-source exsanguination, medication or medical condition-related coagulopathy which could not be easily reversed within 30 min of consultation, pseudoaneurysm infection, and overlying soft tissue or skin ischemia.

### Case 1

**Fig. 1:**
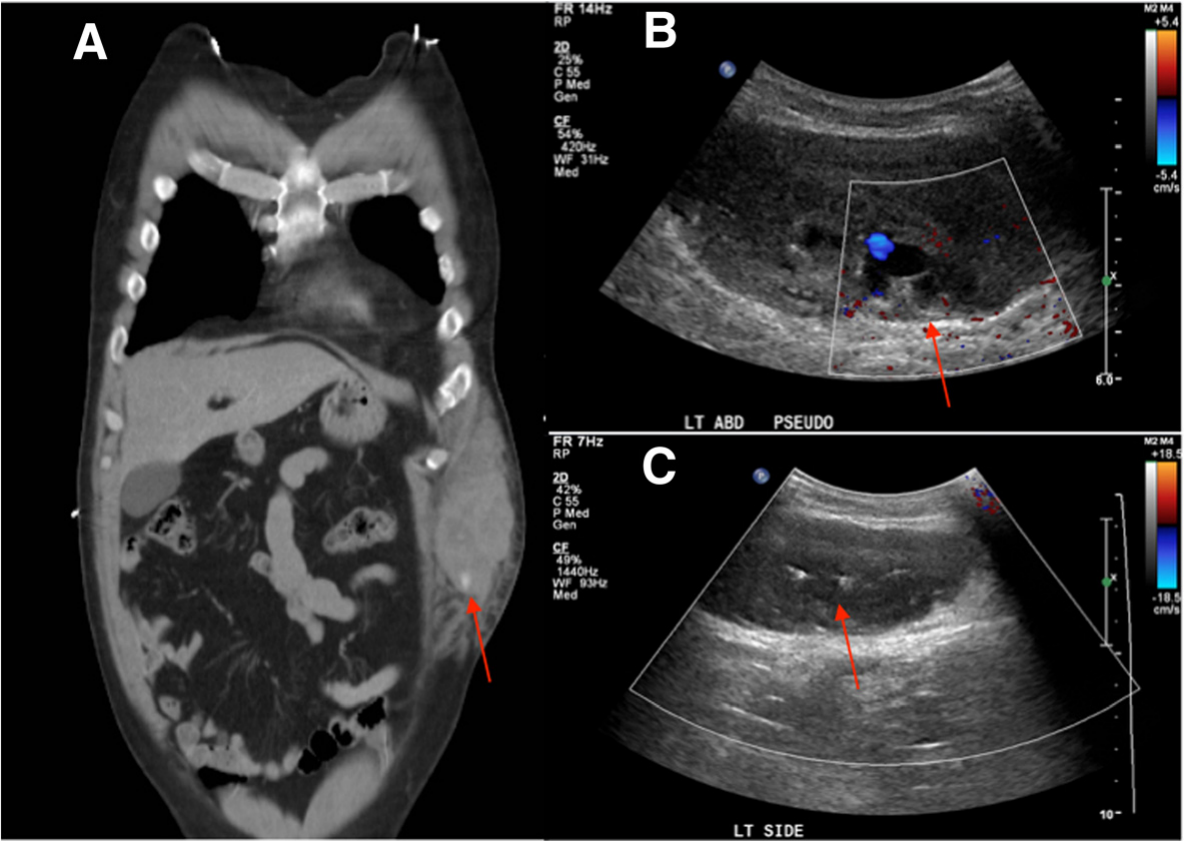
**a** Contrast enhanced CT of the abdomen and pelvis demonstrates a large left anterior abdominal wall hematoma with an area of active extravasation. **b** Ultrasound images with color Doppler through the left flank hematoma demonstrates focal pseudoaneurysm corresponding to the focus of active hemorrhage on the CT. **c** Short term follow up ultrasound image with color Doppler through the left flank hematoma demonstrates no residual pseudoaneurysm

A 60-year-old male with history of diabetes mellitus type 2 and coronary artery disease status posting stenting and maintenance anticoagulation presented with progressively worsening abdominal wall swelling after falling through the rafters in his attic earlier the same day. Physical examination demonstrated an enlarged left anterior abdominal wall hematoma with additional sites of ecchymosis along the pelvic sidewalls and gluteal regions. Pertinent medications included daily aspirin 81 mg and Plavix 75 mg. CT examination demonstrated a large left rectus sheath hematoma with active extravasation, presumably from the left inferior epigastric artery (Fig. 1a). Interventional radiology consultation was immediately sought. Upon confirmation of hemodynamic stability, the patient was considered a good candidate for initial assessment with color Doppler ultrasound examination of the left anterior abdominal wall, confirming the presence of a pseudoaneurysm (Fig. 1b). Under ultrasound guidance, 1000 units of thrombin were infused into the pseudoaneurysm using a 25-gauge needle until cessation of flow was observed (Fig. 1c). The patient tolerated the procedure well, and was discharged home the following day.

### Case 2

**Fig. 2:**
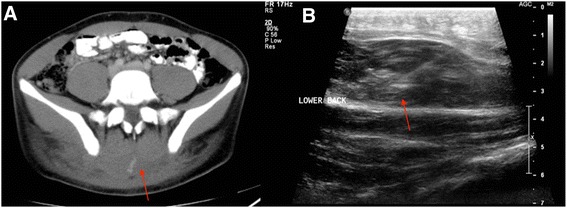
**a** Contrast enhanced CT of the abdomen and pelvis demonstrates a large midline paraspinal hematoma with areas of active extravasation. **b** Ultrasound image through the midline parasagittal hematoma demonstrating needle tip within the hematoma after thrombin injection

A 17-year-old male status post recent motorcycle collision was transferred to our facility for interventional radiologic management of a progressively enlarging region of ecchymosis in the lower back. CT evaluation confirmed the presence of a large midline paraspinal hematoma with a focal zone of contrast blush concerning for either extravasation or pseudoaneurysm formation (Fig. 2a). As the patient was hemodynamically stable at the time of IR consultation, sonographic assessment with color Doppler was performed. The study confirmed a well-defined subcutaneous mid-paraspinal hematoma with central pseudoaneurysm formation. A 25-gauge needle was inserted into the pseudoaneurysm for injection of 1000 units of thrombin. Follow-up imaging confirmed closure of the pseudoaneurysm (Fig. 2b). The patient tolerated the procedure well, and was discharged home the following day.

### Case 3

**Fig. 3:**
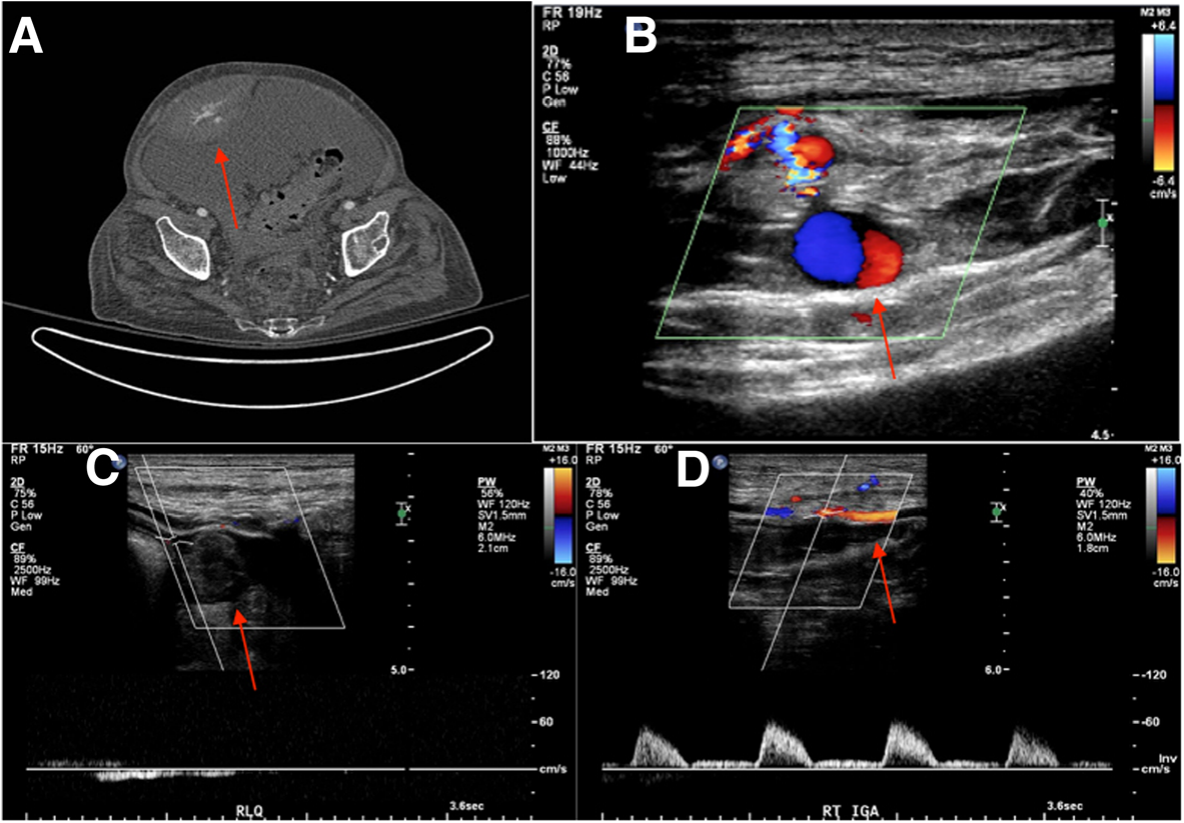
**a** CT angiography of the abdomen and pelvis demonstrates an area of active contrast extravasation within a large right rectus sheath hematoma. **b** Color Doppler ultrasound of the area of active contrast extravasation on the prior CT angiogram demonstrates turbulent color flow in a pattern consistent with a pseudoaneurysm. **c** Follow up ultrasound after thrombin injection into the pseudoaneurysm demonstrates persistent occlusion of the pseudoaneurysm. **d** There is preserved patency of the right inferior epigastric artery

A 73-year-old man with a history of Hepatitis C cirrhosis, hepatocellular carcinoma, and lymphoma as well as diabetes mellitus and chronic kidney disease secondary to hepatorenal syndrome was admitted with malaise, abdominal discomfort, and elevated ammonia and creatinine. During the patient’s hospital course, therapeutic paracentesis procedures were frequently performed for recurrent abdominal ascites-related distension. During a paracentesis attempt on hospital day 8, the procedure was halted after pulsatile bleeding into the right rectus sheath was identified in the region of the right inferior epigastric artery. A CT angiogram of the abdomen and pelvis was performed, which confirmed an area of active contrast extravasation (Fig. 3a). Interrogation of the area of extravasation identified on CT with color Doppler ultrasound revealed a pseudoaneurysm (Fig. 3b). Under ultrasound guidance, 1000 units of thrombin were injected into the pseudoaneurysm using a 25-gauge needle until vascular flow was no longer identified (Fig. 3c). Follow up evaluation of the inferior epigastric artery and pseudoaneurysm demonstrated persistent occlusion of the pseudoaneurysm with maintained vascular flow in the remaining inferior epigastric artery (Fig. 3d). The patient tolerated the procedure well, and was discharged home following management of his remaining medical co-morbidities.

### Case 4

**Fig. 4:**
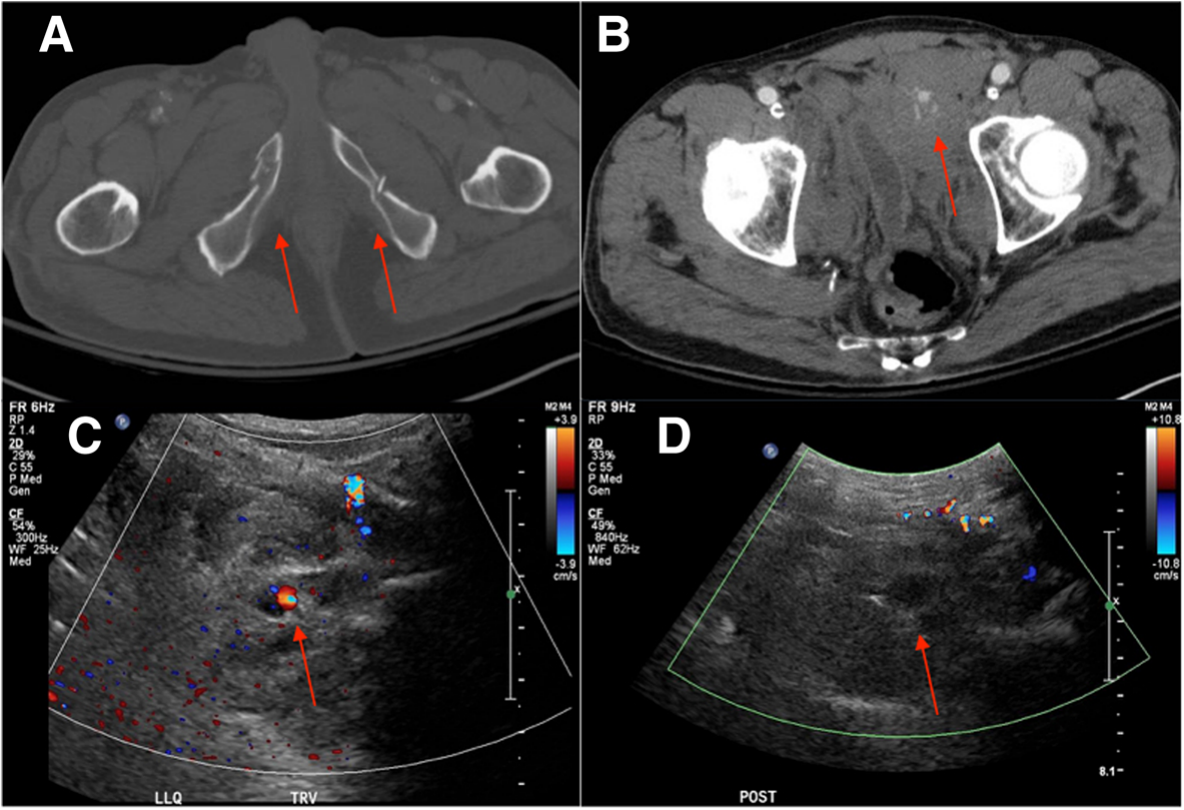
**a** and **b** Contrast enhanced CT of the pelvis demonstrates fractures of the bilateral pubic rami and areas of active hemorrhage likely arising from left external pudendal artery. **c** Color Doppler ultrasound image through the prevesical hematoma demonstrates focal pseudoaneurysm formation corresponding to the area of extravasation on the CT. **d** Following injection of 2500 units of thrombin, no residual vascular flow was identified in the pseudoaneurysm

A 62-year-old man with a history of an aortobifemoral bypass presented as a trauma following a fall from a stepladder with reported pain on the right side. A survey by the trauma clinicians revealed the patient to be hypertensive with tenderness to palpation at the right hip and sacrum. A contrast enhanced CT was performed that demonstrated multiple pelvic fractures, including fractures of the bilateral pubic rami (Fig. 4a). There was an associated large left prevesical space hematoma demonstrating active hemorrhage, appearing to arise from the left external pudendal artery (Fig. 4b). Given the patient’s history of aortic bypass and extensive vascular disease, access to the suspected bleeding vessel was limited. In this setting, ultrasound-guided injection of 2500 units of thrombin using a 25-gauge needle was performed. Two days after the procedure, follow up imaging demonstrated stability of the hematoma size without evidence of active hemorrhage. The patient tolerated the procedure well, and was discharged home following management of his remaining medical co-morbidities.

### Case 5

**Fig. 5:**
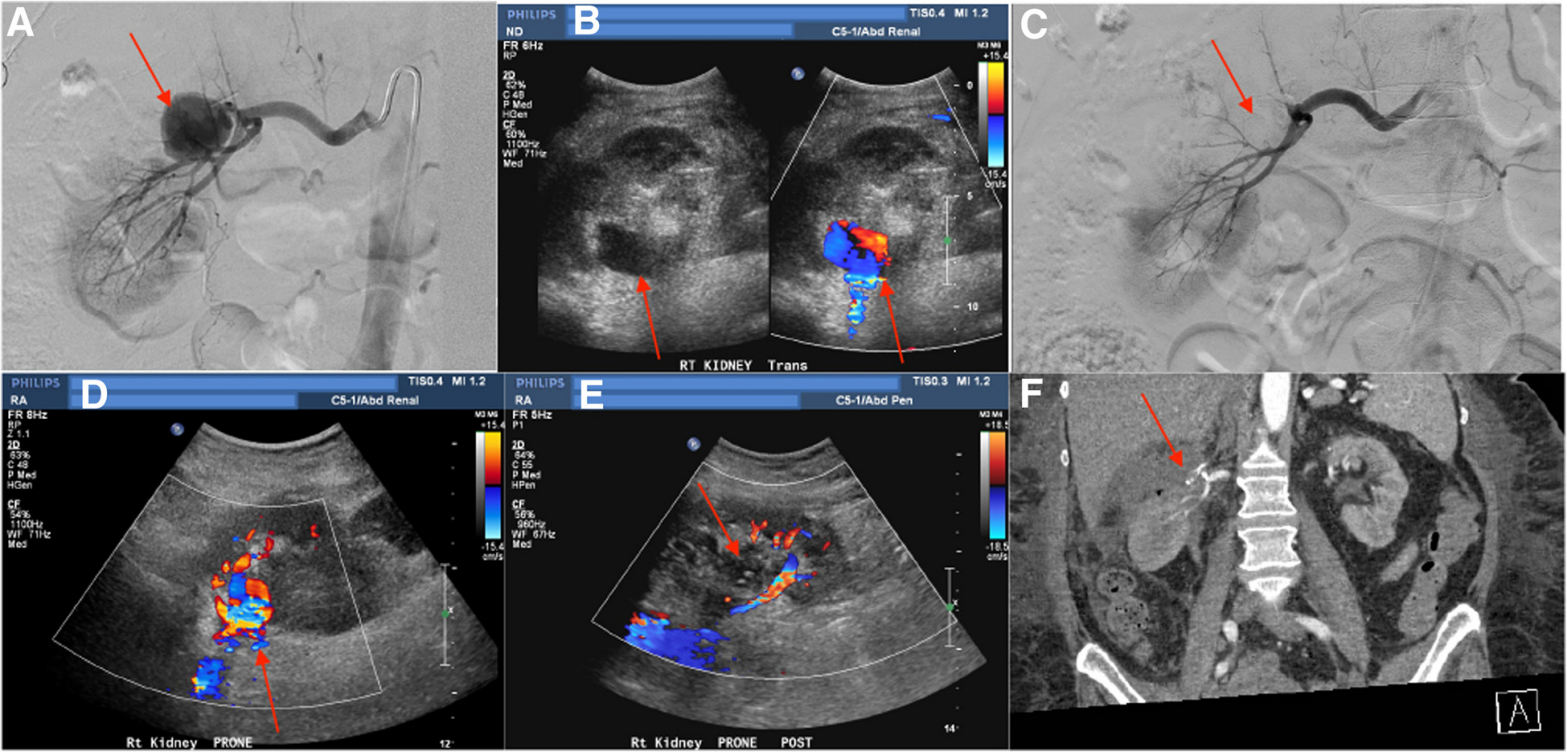
**a** Right renal artery angiogram demonstrates a large pseudoaneurysm arising from an interpolar branch of the right renal artery. **b** Renal sonography with color Doppler performed 3 days following glue embolization demonstrates persistent flow to the pseudoaneurysm. **c** Repeat renal angiography fails to demonstrate the residual pseudoaneurysm.**d** and **e** Color Doppler images of the right kidney before and following administration of 200 units of thrombin confirm no vascular flow in the pseudoaneurysm post-injection. **f** CTA examination performed 2 months following ultrasound-guided thrombin injection confirms occlusion of the pseudoaneurysm

A 73-year-old female status post partial right nephrectomy for management of renal cell carcinoma presented 2 weeks following the procedure with hemodynamically significant hematuria. Upon discovery of a 3 cm right renal pseudoaneurysm along the nephrectomy plane, the patient was referred to the interventional radiology service for management. Emergent angiographic evaluation confirmed the large pseudoaneurysm arising from an interpolar branch of the right renal artery (Fig. 5a). Following n-BCA glue embolization, repeat angiography showed apparent closure of the pseudoaneurysm. However, the patient’s hematuria persisted post embolization, eventually requiring continuous bladder irrigation. Renal sonography performed 2 days later demonstrated persistence of the right pseudoaneurysm (Fig. 5b). The patient was taken back to the interventional suite for repeat embolization. Catheterization of the pseudoaneurysm was unsuccessful given difficulty in accessing the feeding artery (Fig. 5c). Given failure of angiographic treatment, percutaneous access under ultrasound guidance was performed. After confirmation of a preserved yet somewhat attenuated “to and fro” flow pattern in the pseudoaneurysm, ultrasound-guided injection of 200 units of thrombin using a 25-gauge needle was done to complete pseudoaneurysm occlusion (Fig. 5d and e). Following the procedure, the patient’s hematuria had subsequently resolved. The patient tolerated the procedure well, and was discharged to rehab following management of her remaining medical co-morbidities. Repeat CTA performed 2 months following the procedure confirmed occlusion of the pseudoaneurysm (Fig. 5f).

## Discussion

Rectus sheath hematomas are anterior abdominal wall bleeds that typically occur as a complication of anticoagulation or traumatic injury to typically the inferior epigastric vasculature. These hemorrhages have the potential to become hemodynamically significant if a substantial amount of blood accumulates in the anterior abdominal wall [11, 12]. A similarly related entity is the formation of pseudoaneurysms. True aneurysms are abnormal areas of arterial dilation involving the intima, media, and adventitia, the three native layers of an artery wall, and are often related to systemic diseases such as hypertension and atherosclerosis. Pseudoaneurysms, in contrast, occur secondary to blunt or penetrating injury to arteries and do not involve equal dilation of the three native vessel layers. Provided blood flow to the focal area of dilation is maintained, pseudoaneurysms continue to grow in size until their eventual rupture and clinically significant exsanguination [13, 14].

An important step in the management of clinically significant hematomas and pseudoaneurysms stems in the cessation of contributing blood flow. CT, MRI, and ultrasound permit non-invasive diagnosis, while catheter-based angiography under fluoroscopic guidance allows for simultaneous imaging and treatment of these conditions. In the setting of trauma, angiography provides excellent characterization of pseudoaneurysms as well as a roadmap for treatment through embolization [11, 15].

Despite the label of being minimally invasive, angiographic procedures have their own inherent risks, including those of vascular injury during catheter and guide-wire manipulation or post-procedural complications related to access site or medical management. In certain cases of superficial trauma, highly selective catheterization of the injured vessel can become difficult, if not impossible. Campaigns such as Image Gently, Step Lightly**®** and Image Wisely**®** have heightened both public and physician awareness of the adverse effects of prolonged ionizing radiation exposure, and have made great strides in establishing the importance of minimizing radiation exposure when medically applicable.

Thrombin-injection for the management of catheter-associated pseudoaneurysm formation in the femoral and subclavian vasculature has been described [16, 17]. Case reports regarding this utilization of ultrasound guidance for pseudoaneurysm treatment outside femoral vasculature treatment have also been published. While several manuscripts exist concerning the closure of iatrogenic pseudoaneurysms, its role in the management of pseudoaneurysm formation from superficial trauma continues to be employed and reported on a case-by-case basis.

Our interventional team carefully reviewed the clinical, laboratory, and imaging parameters of a given patient prior to consideration for percutaneous thrombin injection. The relative contraindications for percutaneous transcatheter embolization put forth by the Society of Interventional Radiology were considered absolute contraindications for ultrasound-guided approach in our study [18]. While this strict selection served as a control to ensure patient safety, this significantly limited our inclusion patient population, with the remaining trauma interventions managed in our department managed during this time period undergoing primary transcatheter embolization.

Suspicion of active extravasation was considered the parameter of primary importance at the time of initial consultation. Appropriately recognizing each patients’ hemodynamic stability status was of paramount importance, as intraoperative and angiographic interventions remain the gold standard for the cessation of hemorrhage in the critically ill patient [19]. Additionally – as demonstrated in cases 4 and 5 – limited vascular access can preclude angiographic intervention. After thorough clinical examination and review of pre-procedural imaging, our interventionalists proceeded with percutaneous thrombin injection of the culprit superficial pseudoaneurysm contributing to surrounding hematoma formation in the described cases.

Injections of thrombin using a 25-gauge needle totaling 1000 units were utilized in three of our five patients. This total dose is routinely used in the treatment of iatrogenic pseudoaneurysms of the SFA [20]. All patients managed by our interventional group undergo outpatient follow-up within two months of their procedure with either our radiologists or the referring physician. Thrombin-injection in each case was efficacious and without clinical complication. There was no need for repeat intervention or surgery in each case.

The decision to stray away from the typical treatment dose depended on the clinical situation. In case 4, a total thrombin dose of 2500 units was used to complete thrombosis of the left external pudendal artery pseudoaneurysm. It was thought that the deep location of the aneurysm (relative to the superficial location of the other three treated lesions) and larger size of its associated hematoma warranted more aggressive intervention. In case 5, a total thrombin dose of 200 units was administered to occlude the renal interpolar pseudoaneurysm. Given that vascular access to the lesion was limited, it was felt that the smaller dose of thrombin was sufficient to ensure pseudoaneurysm closure.

The time to achieve clinically evident thrombosis in our 5 cases was minimum 12 min, maximum 34 min, mean of 22 min. This was in compared to our typical angiographic cases for traumatic pseudoaneurysm management, with typical departmental procedural times ranging between 40 min minimum up to 120 min.

In summary, ultrasound-guided procedures are minimally invasive and able to achieve the diagnostic and therapeutic efficacy of its angiographic equivalent at the hands of skilled intervenionalists. The described cases show how ultrasound-guided thrombin injection can be a method of primary treatment in otherwise clinically stable patients, or an alternative treatment in cases where transcatheter embolization fails. Although long-term follow-up is needed to further validate our conclusions, this technique has the potential to develop into the mainstay management option of traumatic pseudoaneurysm development in nearly any ultrasound accessible artery.
